# Supplementary material for: Application of fused graphical lasso to statistical inference for multiple sparse precision matrices
Source: PLoS One. 2024 May 31;19(5):e0304264. doi: 10.1371/journal.pone.0304264 (PMC11142621; doi:10.1371/journal.pone.0304264)
Supplement: Attachment — Submitted filename: PONE-D-23-38136-review.pdf [file pone.0304264.s001.pdf]

Review of PONE-D-23-38136

The authors study the problem of estimating multiple precision matrices from multiple populations. They propose to use the fused graphical lasso (FGL) method, which is a lasso penalty on sparsity of precision matrices plus a refined lasso penalty on the two precision matrices that restrains the similar structure across multiple groups. They obtain some inequalities for multiple estimators of FGL models in terms of L_1 and Frobenius matrix norms under several conditions. Built on the work of Jankova and van de Geer (2015) who investigated a de-biasing technology to obtain a new consistent estimator with known distribution, the authors extend the statistical inference problem to multiple populations, and propose the de-biasing FGL estimators. The corresponding asymptotic property of de-biasing FGL estimators is provided. A simulation study and an application of the diffuse large B-cell lymphoma data demonstrate theoretical results. It is a nice contribution. Some comments are below.

1. I noticed that this paper is the same as the one in the ArXiv with id number “arXiv:2303.01326‘”, but the author (Zhidong Bai) does not appear in the submission version. Please give some explanations.

2. The authors list some conditions including Conditions 1 and 2 as well as many restrictions on parameters such as λ and λ_0 in theorems. These assumptions guarantee the results in theorems. Can authors provide some examples to illustrate the suitability of these conditions or restrications? Weak or Strong?

3. Theorem 5 states a limiting null distribution for testing the equality of some entry in two precision matrices. Can this result be extended into multiple precision matrices?

4. Zhang et al. (2021) studied a joint estimation problem of precision matrices for multiple populations with a different method. It is related to your work and could be cited.

References

Zhang, X.-F., Ou-Yang, L., Yan, T., Hu, X. T., and Yan, H. (2021). A joint graphical model for inferring gene networks across multiple subpopulations and data types. *IEEE Transactions on Cybernetics*, 51(2):1043–1055.
