# Supplementary material for: Application of fused graphical lasso to statistical inference for multiple sparse precision matrices
Source: PLoS One. 2024 May 31;19(5):e0304264. doi: 10.1371/journal.pone.0304264 (PMC11142621; doi:10.1371/journal.pone.0304264)
Supplement: Attachment — Submitted filename: Response to Reviewers_PONE_D_23_38136.pdf [file pone.0304264.s002.pdf]

Responses to the comments from Reviewer I

Thanks for your careful reading of our paper. Below we provide our responses to your insightful and constructive comments one by one.

1. **I noticed that this paper is the same as the one in the ArXiv with id number "arXiv:2303.01326", but the author (Zhidong Bai) does not appear in the submission version. Please give some explanations.**
   Response:
   —Thank you very much for your insightful and constructive suggestion.
   —Professor Bai came up with some suggestions during the manuscript writing period. Thus, we submitted the manuscript on ArXiv with author Zhidong Bai appearing on it. However, Professor Bai thought he made little contribution to this paper. So we removed his name with his consent, when we submitted the manuscript to PLoS One.
2. **The authors list some conditions including Conditions 1 and 2 as well as many restrictions on parameters such as λ and λ_0 in theorems. These assumptions guarantee the results in theorems. Can authors provide some examples to illustrate the suitability of these conditions or restrications? Weak or Strong?**
   Response:
   —Thanks for your suggestion.
   —Both Condition 1 and Condition 2 represent weak assumptions of the proposed theorem. Firstly, we assume that

   Condition 1 (Bounded eigenvalues)**.** *There exist universal constants L for k such that*

   $$0 < L < \Lambda_{\min}(\Theta_0^{[k]}) \leq \Lambda_{\max}(\Theta_0^{[k]}) < 1/L < \infty.$$

   This condition is satisfied if the precision matrix is positive definite and its eigenvalues do not fluctuate. It is a weak condition. For example, if the precision matrix fluctuates, we can normalize the original data to ensure that both the covariance matrix and the precision matrix remain are stable. Moreover, after normalization, the eigenvalues of the population covariance matrix will be controlled within a certain range. Consequently, it becomes easier to maintain the eigenvalues of the precision matrix within the set $(L, 1/L)$.
   —Secondly, we assume that

   Condition 2 (Sub-Gaussianity vector condition)**.** *The observations $\mathbf{x}_i^{[k]}$, $i = 1, \ldots, n_k$, are uniformly sub-Gaussian vectors in the respective groups.*

   This is also a weak condition. As for Sub-Gaussian assumption, it is a generalization of the assumption of the Gaussian random variable. Here we mainly require a Sub-Gaussian tail probability. In addition to the Gaussian case, the class of Sub-Gaussian variables includes any bounded random variable (e.g., Bernoulli, multinomial, uniform), any random variable

with strictly log-concave density [6, 20], and any finite mixture of Sub-Gaussian variables. The Sub-Gaussian condition allows more distributions to be included.

—Finally, restrictions on parameters such as λ_0 and λ are also weak. The distance between the sample covariance matrix and the true value can always be controlled by a bound in probability, since the eigenvalues of population covariance matrix are bounded. After data preprocessing, it is easy to set up λ_0, and λ, where λ is constrained by parameter λ_0. In addition, when we prove that the remainder converges to zero in probability in the central limit theorem, the constraint relationship between the parameters is also used.

3. **Theorem 5 states a limiting null distribution for testing the equality of some entry in two precision matrices. Can this result be extended into multiple precision matrices?**

Response:

—Thank you for pointing out this issue.

—In fact, this result can be generalized to multiple precision matrix testing problems. For multiple populations, the concerned hypothesis problem is testing the linear combination of entries on the same location of several different precision matrices, i.e.,

$$H_0 : a_1\Theta_{0ij}^{[1]} + ... + a_K\Theta_{0ij}^{[K]} = 0 \quad vs. \quad H_1 : \text{not } H_0. \tag{1}$$

where $\Theta_{0ij}^{[1]}, ..., \Theta_{0ij}^{[K]}$ are population precision matrices, and $a_1, ..., a_K$ are known constants. We use test statistic

$$a_1\widehat{\Theta}_{dij}^{[1]} + ... + a_K\widehat{\Theta}_{dij}^{[K]}, \tag{2}$$

where $\widehat{\Theta}_{dij}^{[k]}$ are de-biasing estimators, i.e.,

$$\widehat{\Theta}_{dij}^{[k]} = \left(2\widehat{\Theta}^{[k]} - \widehat{\Theta}^{[k]}\widehat{\Sigma}^{[k]}\widehat{\Theta}^{[k]}\right)_{ij}.$$

Corollary 1 shows the limiting distribution of statistic (2) under the null hypothesis. For example, if we consider a three-sample precision matrix testing

$$H_0 : -0.6\Theta_{0ij}^{[1]} - 0.9\Theta_{0ij}^{[2]} + \Theta_{0ij}^{[3]} = 0 \quad vs. \quad H_1 : \text{not } H_0, \tag{3}$$

the constructed statistic is $-0.6\widehat{\Theta}_{dij}^{[1]} - 0.9\widehat{\Theta}_{dij}^{[2]} + \widehat{\Theta}_{dij}^{[3]}$. To be honest, the limiting distribution of proposed test under null hypothesis depends on the coefficients $a_1, ..., a_K$. Hence, the values of $a_1, ..., a_K$ need to be known in advance. If we test the hypothesis

$$H_0 : \Theta_{0ij}^{[1]} = \Theta_{0ij}^{[2]} = \Theta_{0ij}^{[3]} \quad vs. \quad H_1 : \text{not } H_0, \tag{4}$$

we need to transform it to the hypothesis based on a linear function of the corresponding entries of precision matrices $\{\Theta_{0ij}^{[k]}\}_{k=1,2,3}$. Whether we use the linear hypothesis $(\Theta_{0ij}^{[1]} - \Theta_{0ij}^{[2]}) + (\Theta_{0ij}^{[2]} - \Theta_{0ij}^{[3]}) = 0$ or $a(\Theta_{0ij}^{[1]} - \Theta_{0ij}^{[2]}) + b(\Theta_{0ij}^{[2]} - \Theta_{0ij}^{[3]}) = 0$ for constants a, b, it is not an equivalent transformation of the null hypothesis H_0 in (4). If a test statistic can be constructed directly used for hypothesis problem (4), this is the statistic we prefer. In current paper, we do not consider to proposing the statistic for testing the equality of several precision matrices. It will be included in our future work, and we will make efforts on it.

4. **Zhang et al. (2021) studied a joint estimation problem of precision matrices for multiple populations with a different method. It is related to your work and could be cited.**
Response:
—Thanks for your suggestion.
—Gene expression data are divided into several subpopulations and data types. In most applications, scholars pay attention to a single gene network from a single dataset, however, Zhang et al. [1] focus on the similarities and differences among different subpopulations and data types. They propose a new joint group lasso penalty to restore the multiple gene networks, which is effective for data with several subpopulations and data types. It is helpful for our work. Following your suggestion, we have quoted this article in revision and marked it in red.

Thank you again for all your insightful comments and helpful suggestions.

Responses to the comments from Reviewer II

Thanks for your careful reading of our paper. Below we provide our responses to your insightful and constructive comments one by one.

1. **As mentioned in paper, author needs to minimize the negative penalized log likelihood function (1) and get the algorithm solution as an estimator in both numerical study and real data application. Author uses the ADMM algorithm to execute the estimating process with classical AIC tuning method. Could the author demonstrate the ADMM algorithm for fused lasso? Will the solutions with different tuning methods affect the accuracy of the hypothesis testing process? Is there any better approach to tune the parameters?**

   Response:

   —Thank you for this nice question.

   —To solve the optimization problem

$$\{\widehat{\Theta}^{[k]}\} = \arg\min_{\{\Theta^{[k]}\in\mathcal{S}^{++}\}} \sum_k \{tr(\widehat{\Sigma}^{[k]}\Theta^{[k]}) - \log\det(\Theta^{[k]})\} + \mathbf{P}(\{\Theta^{[k]}\}), \tag{5}$$

   with fused lasso penalty

$$\mathbf{P}(\{\Theta^{[k]}\}) = \lambda\sum_{k=1}^{K} ||(\Theta^{[k]})^{-}||_1 + \rho\sum_{k<k'} ||(\Theta^{[k]} - \Theta^{[k']})^{-}||_1, \tag{6}$$

   we give the ADMM algorithm for fused lasso as follows. We first re-write it as

$$\begin{aligned} &\text{minimize} \quad \sum_k \{tr(\widehat{\Sigma}^{[k]}\Theta^{[k]}) - \log\det(\Theta^{[k]})\} + \mathbf{P}(\{\Theta^{[k]}\}) \\ &\text{subject to} \quad \Theta^{[k]} = \mathbf{Z}^{[k]}, \quad \Theta^{[k]} - \Theta^{[k+1]} = \Delta^{[k]}. \end{aligned} \tag{7}$$

   where $\mathbf{Z}^{[k]}$ and $\Delta^{[k]}$ are the slack variables. The augmented Lagrangian for this problem is

$$\begin{aligned} &\mathbf{L}\left(\{\Theta^{[k]}\},\{\mathbf{Z}^{[k]}\},\{\Delta^{[k]}\},\{\mathbf{V}^{[k]}\},\{\mathbf{U}^{[k]}\}\right) \\ &= \sum_k \{tr(\widehat{\Sigma}^{[k]}\Theta^{[k]}) - \log\det(\Theta^{[k]})\} \\ &+ \sum_{k=1}^{K}\left\{\lambda_1||(\mathbf{Z}^{[k]})^{-}||_1 + (\rho_1/2)||(\Theta^{[k]} - \mathbf{Z}^{[k]} + \mathbf{V}^{[k]})^{-}||_F^2 - (\rho_1/2)||(\mathbf{V}^{[k]})^{-}||_F^2\right\} \\ &+ \sum_{k=1}^{K-1}\left\{\lambda_2||(\Delta^{[k]})^{-}||_1 + (\rho_2/2)||(\Theta^{[k]} - \Theta^{[k+1]} - \Delta^{[k]} + \mathbf{U}^{[k]})^{-}||_F^2 - (\rho_2/2)||(\mathbf{U}^{[k]})^{-}||_F^2\right\}, \end{aligned} \tag{8}$$

   where $\mathbf{V}^{[k]}$ and $\mathbf{U}^{[k]}$ are the scaled dual variable associated with the constraint, and ρ_1 and ρ_2 are the positive penalty parameters, $||\cdot||_F$ denotes Frobenius norm. If $k = 1$, denotes

$$\mathbf{Y}^{[1]}(\mathbf{V}^{[1]},\mathbf{Z}^{[1]},\mathbf{U}^{[1]},\Delta^{[1]}) = \widehat{\Sigma}^{[1]} + \rho_1\left(\mathbf{V}^{[1]} - \mathbf{Z}^{[1]}\right) + \rho_2\left(\mathbf{U}^{[1]} - \Delta^{[1]} - \Theta^{[2]}\right); \tag{9}$$

if $1 < k < K$, denotes

$$\begin{aligned}&\mathbf{Y}^{[k]}(\mathbf{V}^{[k]}, \mathbf{Z}^{[k]}, \mathbf{U}^{[k]}, \Delta^{[k]}) \\ &= \widehat{\Sigma}^{[k]} + \rho_1 \left(\mathbf{V}^{[k]} - \mathbf{Z}^{[k]}\right) - \rho_2 \left(\mathbf{U}^{[k-1]} - \Delta^{[k-1]} + \Theta^{[k-1]}\right) + \rho_2 \left(\mathbf{U}^{[k]} - \Delta^{[k]} - \Theta^{[k+1]}\right);\end{aligned} \tag{10}$$

otherwise,

$$\mathbf{Y}^{[K]}(\mathbf{V}^{[K]}, \mathbf{Z}^{[K]}, \mathbf{U}^{[K]}, \Delta^{[K]}) = \widehat{\Sigma}^{[K]} + \rho_1 \left(\mathbf{V}^{[K]} - \mathbf{Z}^{[K]}\right) - \rho_2 \left(\mathbf{U}^{[K-1]} - \Delta^{[K-1]} + \Theta^{[K-1]}\right). \tag{11}$$

A piecewise function $m_\alpha(z)$ of a variable z with parameter α is defined as

$$m_\alpha(z) = \begin{cases} z + \alpha, & z < -\alpha \\ 0, & |z| < \alpha \\ z - \alpha, & z > \alpha, \end{cases} \tag{12}$$

and the function $M_\alpha(Z)$ of a matrix Z returns a matrix, i.e. $M_\alpha(Z) = \left(m_\alpha(Z_{ij})\right)_{i,j}$. Let $\mathrm{Y}^{[k]}$ be the off-diagonal matrix of $\mathbf{Y}^{[k]}$. The update rules are

$$\Theta^{[k]}_{(i_t)} \leftarrow \begin{cases} \frac{1}{2(\rho_1+\rho_2)} \left[-\mathrm{Y}^{[k]}_{(i_t-1)} + \sqrt{(\mathrm{Y}^{[k]}_{(i_t-1)})'\mathrm{Y}^{[k]}_{(i_t-1)} + 4(\rho_1 + \rho_2)\mathbf{I}}\right] & k = 1, K \\ \frac{1}{2(\rho_1+2\rho_2)} \left[-\mathrm{Y}^{[k]}_{(i_t-1)} + \sqrt{(\mathrm{Y}^{[k]}_{(i_t-1)})'\mathrm{Y}^{[k]}_{(i_t-1)} + 4(\rho_1 + 2\rho_2)\mathbf{I}}\right] & 1 < k < K. \end{cases} \tag{13}$$

$$\mathbf{Z}^{[k]}_{(i_t)} \leftarrow \left(M_{\frac{\lambda_1}{\rho_1}}(\Theta^{[k]}_{(i_t)} + \mathbf{V}^{[k]}_{(i_t-1)})\right)^{-} \tag{14}$$

$$\Delta^{[k]}_{(i_t)} \leftarrow \left(M_{\frac{\lambda_2}{\rho_2}}(\Theta^{[k]}_{(i_t)} - \Theta^{[k+1]}_{(i_t)} + \mathbf{U}^{[k]}_{(i_t-1)})\right)^{-} \tag{15}$$

$$\mathbf{V}^{[k]}_{(i_t)} \leftarrow \left(\mathbf{V}^{[k]}_{(i_t-1)} + \Theta^{[k]}_{(i_t)} - \mathbf{Z}^{[k]}_{(i_t)}\right)^{-} \tag{16}$$

$$\mathbf{U}^{[k]}_{(i_t)} \leftarrow \left(\mathbf{U}^{[k]}_{(i_t-1)} + \Theta^{[k]}_{(i_t)} - \Theta^{[k+1]}_{(i_t)} - \Delta^{[k]}_{(i_t)}\right)^{-}, \tag{17}$$

where i_t is the iteration of the algorithm. From the analysis above, we note that the positive definiteness constraint on the precision matrices is naturally enforced by the update (13). In our algorithm, the stopping criterion is

$$\frac{\sum_{k=1}^{K} ||\widehat{\Theta}^{[k]}_{(i_t)} - \widehat{\Theta}^{[k]}_{(i_t-1)}||_1}{\sum_{k=1}^{K} ||\widehat{\Theta}^{[k]}_{(i_t-1)}||_1} \leq \varepsilon, \tag{18}$$

where $\varepsilon = 10^{-9}$, and $\widehat{\Theta}^{[k]}_{(i_t)}$ denotes the estimate of $\Theta^{[k]}_{(i_t)}$ in the i_t-th step of the algorithm. **Algorithm** 1 represents the iterative process for multiple populations FGL model.

—We have tried different tuning methods, and we obtained the similar test results with these different tuning methods. Thus, tuning method doesn't affect the accuracy of the hypothesis testing process. However, it affects the tuning time. If the similar testing result can be obtained through a faster tuning method, this tuning method is the one we prefer. This will be included in our future work.

Algorithm 1 Estimating parameters of the fused graphical lasso.

Require:

Loading data set $\mathbf{X}^{[k]}$, and the non-negative parameters λ_1, ρ_1, λ_2, and ρ_2.

Ensure:

1: Initialization: $\widehat{\Theta}^{[k]}_{(0)} = (\widehat{\Sigma}^{[k]} + 0.1\mathbf{I})^{-1}$, $\mathbf{Z}^{[k]}_{(0)} = \widehat{\Theta}^{[k]}_{(0)}$, $\boldsymbol{\Delta}^{[k]}_{(0)} = \widehat{\Theta}^{[k]}_{(0)} - \widehat{\Theta}^{[k+1]}_{(0)}$, $\mathbf{V}^{[k]}_{(0)} = \mathbf{1}_p\mathbf{1}'_p$, and $\mathbf{U}^{[k]}_{(0)} = \mathbf{1}_p\mathbf{1}'_p$;

2: While iteration $<$ maximize iteration:

3: In the i_t-th iteration, given the estimated $\widehat{\Theta}^{[k]}_{(i_t)}$, updating (13)-(17);

4: Repeat until convergence.

2. **In page 2, line 13: "$\hat{\Sigma}^{-1}$" $\rightarrow$ "$\hat{\Sigma}_n^{-1}$".**

   Response:

   —We have already corrected and marked it in red in revision, thank you.

3. **In page 4, line 2: "we denote $(A)_{ij}$ its (i, j)-entry" $\rightarrow$ "we denote $(A)_{ij}$ as (i, j)-entry of A".**

   Response:

   —Corrected, thank you.

4. **In page 4, line 3 and line 18: Author confused the symbols of the determinant and the cardinality, please distinguish them.**

   Response:

   —In the revision, we have redefined the two concepts and used different notations to distinguish them. We write $\big|A\big|$ for the determinant of matrix A, and $|S|$ denotes the cardinality of the set S. Thank you for your comments.

5. **In page 11, line 17: what does notation $|A|$ mean? Please clarify the meaning of the symbol.**

   Response:

   —In the context, it represents the cardinality of a set A. Again, we redefined the definition of determinant of a matrix, and modified it. Thus, we can distinguish the determinant of a matrix and the cardinality of a set in the revision. Thank you for your valuable comments.

6. **There are too many formulas with numbers. Please remove the numbers of formulas that are not used.**

   Response:

   —Too many labels did cause the ambiguity. We have already got rid of the useless labels, thank you.

Thank you again for all your insightful comments and helpful suggestions.

References

[1] Zhang, X.-F., Ou-Yang, L., Yan, T., Hu, X. T., Yan, H., 2019. A joint graphical model for inferring gene networks across multiple subpopulations and data types. IEEE Transactions on Cybernetics 51 (2), 1043–1055.
